# A comparison of transcranial Doppler with near infrared spectroscopy and indocyanine green during hemorrhagic shock: a prospective experimental study

**DOI:** 10.1186/cc3980

**Published:** 2006-01-23

**Authors:** Berthold Bein, Patrick Meybohm, Erol Cavus, Peter H Tonner, Markus Steinfath, Jens Scholz, Volker Doerges

**Affiliations:** 1 Medical Doctor, Department of Anaesthesiology and Intensive Care Medicine, University Hospital Schleswig-Holstein, Campus Kiel, Germany; 2 Resident, Department of Anaesthesiology and Intensive Care Medicine, University Hospital Schleswig-Holstein, Campus Kiel, Germany; 3 Professor of Anaesthesiology and Vice-Chair, Department of Anaesthesiology and Intensive Care Medicine, University Hospital Schleswig-Holstein, Campus Kiel, Germany; 4 Professor of Anaesthesiology, Department of Anaesthesiology and Intensive Care Medicine, University Hospital Schleswig-Holstein, Campus Kiel, Germany; 5 Professor of Anaesthesiology and Chair, Department of Anaesthesiology and Intensive Care Medicine, University Hospital Schleswig-Holstein, Campus Kiel, Germany

## Abstract

**Introduction:**

The present study was designed to compare cerebral hemodynamics assessed using the blood flow index (BFI) derived from the kinetics of the tracer dye indocyanine green (ICG) with transcranial Doppler ultrasound (TCD) in an established model of hemorrhagic shock.

**Methods:**

After approval from the Animal Investigational Committee, 20 healthy pigs underwent a simulated penetrating liver trauma. Following hemodynamic decompensation, all animals received a hypertonic-isooncotic hydroxyethyl starch solution and either arginine vasopressin or norepinephrine, and bleeding was subsequently controlled. ICG passage through the brain was monitored by near infrared spectroscopy. BFI was calculated by dividing maximal ICG absorption change by rise time. Mean blood flow velocity (FVmean) of the right middle cerebral artery was recorded by TCD. FVmean and BFI were assessed at baseline (BL), at hemodynamic decompensation, and repeatedly after control of bleeding.

**Results:**

At hemodynamic decompensation, cerebral perfusion pressure (CPP), FVmean and BFI dropped compared to BL (mean ± standard deviation; CPP 16 ± 5 mmHg versus 70 ± 16 mmHg; FVmean 4 ± 5 cm·s^-1 ^versus 28 ± 9 cm·s^-1^; BFI 0.008 ± 0.004 versus 0.02 ± 0.006; *p *< 0.001). After pharmacological intervention and control of bleeding, FVmean and BFI increased close to baseline values (FVmean 23 ± 9 cm·s^-1^; BFI 0.02 ± 0.01), respectively. FVmean and BFI were significantly correlated (r = 0.62, *p *< 0.0001).

**Conclusion:**

FVmean and BFI both reflected the large variations in cerebral perfusion during hemorrhage and after resuscitation and were significantly correlated. BFI is a promising tool to monitor cerebral hemodynamics at the bedside.

## Introduction

Reliable monitoring of cerebral oxygenation is an issue of paramount importance in anesthesia and critical care, since an impaired balance of oxygen demand and supply puts viable brain tissue at risk of ischemia [1]. Cerebral oxygenation is, among other influencing factors, highly dependent on cerebral blood flow (CBF). Despite its clinical relevance, a reliable and suitable method for measuring CBF rapidly, repeatedly and non-invasively at the bedside is currently still lacking. Perfusion magnetic resonance and computed tomographic imaging, though offering a very high spatial resolution, are both limited by the fact that they are not suitable for point of care monitoring and, therefore, cannot provide repeated measurements [2]. Transcranial Doppler ultrasound (TCD) has been advocated as a bedside monitor of CBF, but is technically challenging and, in a notable proportion of patients, a sufficient ultrasound window is lacking [3]. Measurement of both jugular venous oxygen saturation and local brain tissue oxygen pressure (ptiO_2_) are invasive techniques and severe complications have been described [4]. Near infrared spectroscopy (NIRS) is a non-invasive technique capable of detecting changes in cerebral oxygenation and cerebral blood volume continuously [5]. NIRS also enables detection of the tracer dye indocyanine green (ICG), which shows an absorption peak at 805 nm, during its passage through the cerebral vasculature after intravenous injection. Rapid clearance from the blood by both hepatic uptake and biliary excretion allow for repetitive measurements even at short time intervals. In a preliminary animal study, a blood flow index (BFI) derived from ICG kinetics was significantly correlated with cortical blood flow, but not with skin blood flow, and the BFI was, therefore, found to be suitable for non-invasive estimation of CBF [6]. More recently, the BFI has been shown to allow for rapid and repeated measurements with good reproducibility at the bedside in paediatric patients in the intensive care unit (ICU) [7], and to indicate regional perfusion differences in patients after middle cerebral artery infarction [8]. Because ICG may be injected by any iv access, BFI has been claimed as an at least minimal invasive procedure for determination of cerebral perfusion [9] and an efficient additional tool for that purpose. The present study was designed to evaluate the BFI during a wide range of both physiological and pathophysiological conditions and to compare it with transcranial Doppler ultrasound, an established method of monitoring cerebral hemodynamics at the bedside.

## Materials and methods

Animal Investigation Committee, and animals were managed in accordance with the American Physiologic Society and institutional guidelines. The study was performed according to Utstein-style guidelines on 20 healthy swine (German domestic pigs) ranging from 12 to 16 weeks of age of either gender and weighing 43 to 48 kg. The pigs were premedicated with azaperone (neuroleptic agent; 8 mg·kg^-1 ^i.m.) and atropine (0.05 mg·kg^-1 ^i.m.) 1 hour before surgery. Anesthesia was induced with a bolus dose of ketamine (2 mg·kg^-1 ^i.v.), propofol (1 to 2 mg·kg^-1 ^i.v.) and sufentanil (0.3 μg·kg^-1 ^i.v.) given via an ear vein. After endotracheal intubation during spontaneous ventilation, the pigs were ventilated using a volume-controlled ventilator (Siemens SV 900C, Erlangen, Germany) with 35% oxygen at 20 breaths per minute at a tidal volume of 8 to 10 ml·kg^-1 ^adjusted to maintain normocapnia (end-tidal CO_2 _from 35 to 40 mmHg) and with a positive end-expiratory pressure of 5 mmHg. Anesthesia was maintained with a continuous infusion of propofol (8 to 10 mg·kg^-1^·h^-1^) and sufentanil (0.3 μg·kg^-1^·h^-1^); paralysis was provided by a continuous infusion of pancuronium (0.1 mg·kg^-1^·h^-1^). Ringer's solution (6 ml·kg^-1^·h^-1^) was administered in the preparation phase using an infusion pump (Infusomat, Braun, Melsungen, Germany). A standard lead II electrocardiogram (ECG) was used to monitor cardiac rhythm. Depth of anesthesia was judged according to blood pressure, heart rate, and bispectral index (BIS*XP*, Aspect Medical Systems, Natick, MA, USA). If cardiovascular variables or BIS indicated a reduced depth of anesthesia, additional propofol and sufentanil was given.

A pulmonary artery catheter (PAC; Edwards Swan Ganz Combo EDV Thermodilution Catheter, Baxter Laboratories, Irvine, CA, USA) was inserted via an 8.5 F introducer in the right internal jugular vein, advanced under continuous pressure recording into wedge position and then connected to a cardiac output (CO) computer system (Vigilance Monitor, Baxter Edwards Critical Care, Irvine, CA, USA). CO was determined by bolus pulmonary artery thermodilution using 10 ml ice cold saline injected in the proximal port of the PAC three times randomly assigned to the respiratory cycle. A 7-F saline filled catheter was advanced into the right femoral artery for monitoring aortic blood pressure and heart rate. Mean arterial blood pressure (MAP) was determined by electronic integration of the aortic blood pressure waveform. Body temperature was maintained between 38.0 and 39.0°C with a heating blanket. Ventilation was monitored using an inspired/expired gas analyzer that measured oxygen and end-tidal carbon dioxide (CO_2_: M-PRESTN; Datex-Ohmeda Inc., Helsinki, Finland). Oxygen saturation was monitored by a continuous pulse oxymeter placed on the ear (M-CAiOV; Datex-Ohmeda Inc.). For measurement of intracranial pressure (ICP) a fiberoptic flexible catheter was inserted (Ventrix, Integra NeuroSciences, Plainsboro, NJ, USA) via a multiluminal probe introducer (Licox IM3.STV, GMS, Kiel, Germany) after drilling a 5.3 mm skull burr hole 10 mm paramedian and 10 mm cranial of the coronal suture. Cerebral perfusion pressure (CPP) was defined as MAP minus ICP (CPP = MAP – ICP). Anticoagulation was achieved with an intravenous bolus injection of heparin (100 I·U·kg^-1^) to prevent intracardiac clot formation.

### Near infrared spectroscopy

**Figure 1:**
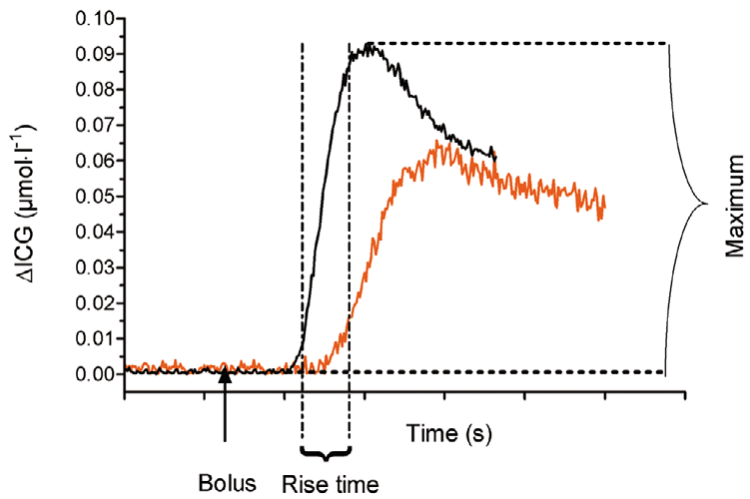
Calculation of the blood flow index. Typical indocyanine green (ICG) measurement in a pig during stable hemodynamics (black line) and at hemodynamic decompensation (orange line).

The NIRO 300 (Hamamatsu Photonics, Herrsching, Germany) is a non-invasive monitor allowing for measurement of concentration changes of the intravascular dye ICG (Pulsion Medical Systems, Munich, Germany). Four wavelengths of light (775, 810, 850, and 910 nm) are delivered by four pulsed laser diodes, and scattered light is detected by three closely placed photodiodes. The specific extinction coefficient of ICG is applied to a modified Beer-Lambert law and absolute concentration changes are calculated by proprietary software (Hamamatsu Photonics). ICG was injected as bolus in the proximal port of the PAC at a dose of 0.1 mg·kg^-1 ^and a concentration of 1 mg·ml^-1^. For each measurement, the time to peak (interval), the rise time (defined as the time between 10% and 90% of the ICG maximum), the slope and the BFI were calculated. The BFI method, originally described by Perbeck and co-workers [10] for blood flow determination in intestinal capillaries, was subsequently applied to ICG dye kinetics in the cerebral vasculature [6]. BFI was calculated as described previously according to the algorithm:



BFI is proportional to blood flow, but the proportionality factor is unknown (Figure 1). This means that BFI measurements are comparable within a subject, but not between subjects, since the proportionality factor may vary considerably between subjects [7].

The optodes of the NIRS were attached to the intact skull covering the right cerebral hemisphere. As increasing the interoptode distance decreases extracerebral contamination, we chose the largest interoptode distance recommended by the manufacturer of our NIRS device. The path length for NIRS measurements was adjusted according to the manufacturer's instructions for measurements on the adult human skull and sampling rate was set to 6 Hz.

### Transcranial Doppler ultrasound

Relative changes of CBF velocity were determined by transcranial Doppler ultrasound (TCD; DWL, Sipplingen, Germany) using the temporal bone window. After removing the overlying skin, the right middle cerebral artery was insonated with a 2 MHz pulsed Doppler probe at a depth of 28 to 32 mm, and mean blood flow velocity (FVmean) was recorded. The transducer was kept fixed in place by an elastic headband to ensure a stable position of vessel insonation.

### Experimental protocol

After taking baseline values, the experiment was started with a midline laparotomy, and an incision was made across the right liver lobe (width, 12 cm; depth, 3 cm, followed by finger fraction) to simulate uncontrolled hemorrhage. Hemodynamic decompensation was defined as a mean arterial pressure of less than 25 mmHg or, since heart rate decreases in the late phase of hemorrhagic shock, a heart rate of less than 20% of its peak value. At that point, the fraction of inspired oxygen (FiO_2_) was raised to 1.0 and all animals received a hypertonic-isooncotic hydroxyethyl starch solution (Hyperhaes^®^, Fresenius, Bad Homburg, Germany; 4 ml·kg^-1 ^over two minutes) and either arginine vasopressin (Pitressin^®^, Parke-Davis, Karlsruhe, Germany, 0.25 IU·kg^-1^) followed by a continuous infusion (2 IU·kg^-1^·h^-1^; 8 animals) or norepinephrine (Aventis Pharma GmbH, Frankfurt am Main, Germany; 25 μg·kg^-1^) followed by a continuous infusion (60 μg·kg^-1^·h^-1^; 12 animals). Bleeding was controlled by manual compression of the liver 30 minutes after drug administration, and FiO_2 _adjusted to 0.5. Crystalloid (Ringer's solution, 10 ml·kg^-1^·h^-1^) and colloid (hydroxyethyl starch 130/0.4, 10 ml·kg^-1^·h^-1^) solutions were administered continuously. NIRS and TCD values were taken during stable baseline conditions, at hemodynamic decompensation, and subsequently 10, 40, and 90 minutes after drug administration. At the end of the experimental protocol, the animals were euthanized with an overdose of propofol, sufentanil and potassium chloride and subjected to necropsy to check for correct positioning of the intravascular catheters.

### Statistical analysis

Statistical comparisons were performed using commercially available statistics software (GraphPad Prism version 4.03 for Windows, GraphPad Software, San Diego, CA, USA). Variables were analyzed with one way repeated measures analysis of variance with Bonferroni correction for multiple comparisons; values are expressed as mean ± standard deviation. Correlation between BFI, TCD, CPP and CO values was analyzed with Spearman's rank correlation. Inter-individual variability of BFI and TCD values was determined by calculating the coefficient of variation (CV) of measurements at each experimental stage. Receiver-operator curves were calculated for a threshold of CPP below 25 mmHg for both interval and rise time. Statistical significance was considered at *p *< 0.05.

## Results

**Figure 2:**
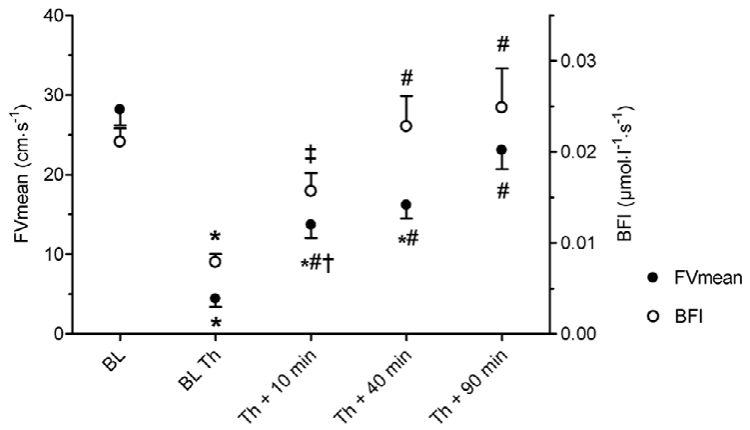
Blood flow velocity by transcranial Doppler ultrasound and bood flow index (BFI) by near infrared spectroscopy at the different experimental stages (BL, baseline; BL Th, start therapy; Th + 10 min, after 10 minutes of therapy; Th + 40 min, after 40 minutes of therapy; Th + 90 min, after 90 minutes of therapy). Data are given as mean ± standard error of the mean; *p < 0.001 versus baseline; ^#^*p *< 0.001 versus baseline therapy; ^‡^*p *< 0.01 versus baseline therapy; ^†^*p *< 0.05 versus therapy + 90 minutes. FVmean, mean blood flow velocity in the right middle cerebral artery.

Hemodynamic data, blood gases and NIRS values at the different experimental stages are presented in Table 1. CV of BFI values was 31%, 49%, 54%, 57% and 55% at baseline, baseline therapy and 10, 40 and 90 minutes after vasopressor administration. CV of TCD measurements at the same experimental stages was 32%, 104%, 51%, 51% and 40%, respectively. Following liver trauma, CPP and CO as well as NIRS and TCD values decreased continuously. At baseline therapy, CPP and CO decreased by 77% and 65%, respectively, whereas heart rate increased by 103% (*p *< 0.001 versus baseline). BFI and FVmean were reduced by 60% and 83% from baseline values, respectively (Figure 2). After vasopressor administration, both CPP and CO increased all along the experimental procedure, reaching 68% and 115% of baseline values 90 minutes after initiation of therapy (*p *< 0.001 versus baseline therapy). BFI and FVmean reflected hemodynamic improvement and were at 78% and 51%, 98% and 65%, and 127% and 93% of baseline values 10, 40 and 90 minutes, respectively, following vasopressor administration (*p *< 0.01 for BFI and *p *< 0.001 for FVmean versus baseline therapy; Figure 2).

**Figure 3:**
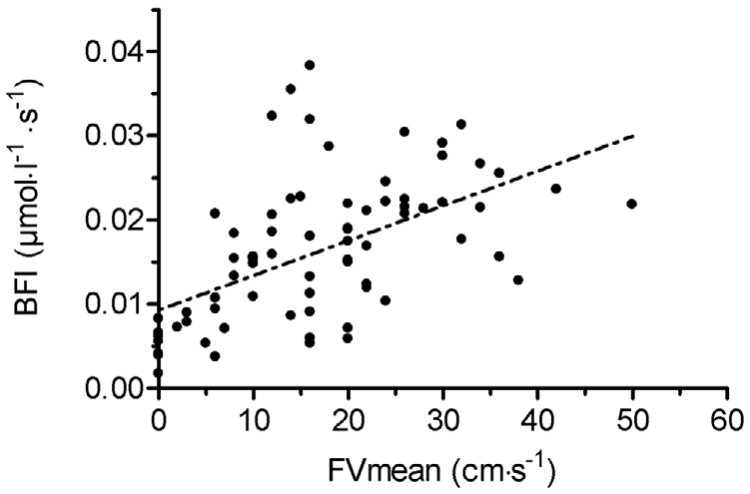
Correlation between blood flow velocity by transcranial Doppler ultrasound and blood flow index (BFI) by near infrared spectroscopy. FVmean, mean blood flow velocity in the right middle cerebral artery; r = 0.62; *p *< 0.0001. n = 76 measurements in 20 animals.

**Figure 4:**
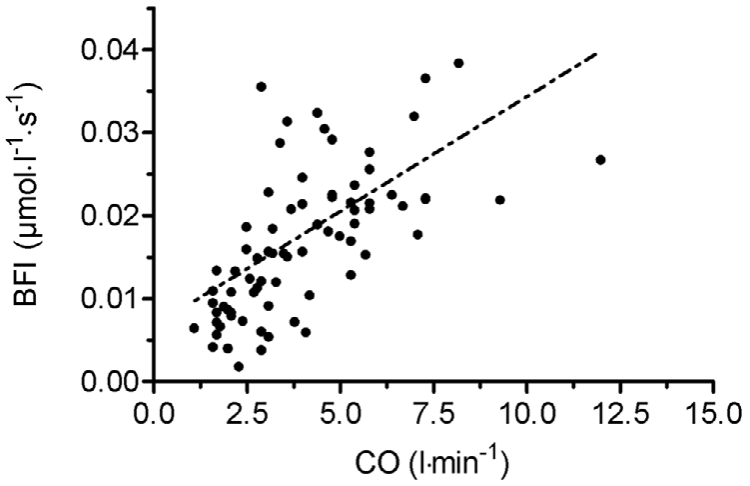
Correlation between cardiac output (CO) and blood flow index (BFI) by near infrared spectroscopy. r = 0.71; *p *< 0.0001. *n* = 76 measurements in 20 animals.

**Figure 5:**
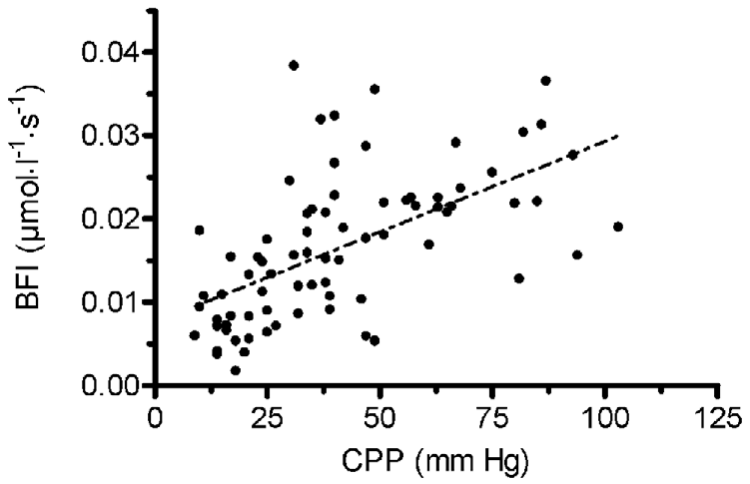
Correlation between cerebral perfusion pressure (CPP) and blood flow index by near infrared spectroscopy. r = 0.66, *p *< 0.0001. *n* = 76 measurements in 20 animals.

BFI was significantly correlated with FVmean (r = 0.62), CPP (r = 0.66) ad CO (r = 0.71) (Figures 3, 4 and 5; *p *< 0.0001). Correlation of slope with FVmean, CPP and CO was 0.59, 0.65 and 0.36, respectively (*p *< 0.0001, *p *< 0.0001 and *p *< 0.001, respectively). Similarly, FVmean was significantly correlated with CPP (r = 0.81, *p *< 0.0001) and CO (r = 0.78, *p *< 0.0001).

**Figure 6:**
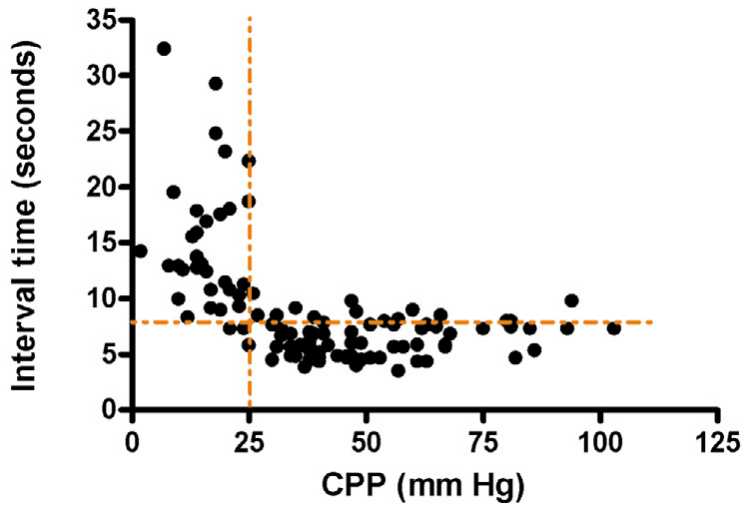
Cerebral perfusion pressure (CPP) and near infrared spectroscopy derived interval time. *n* = 76 measurements in 20 animals.

**Figure 7:**
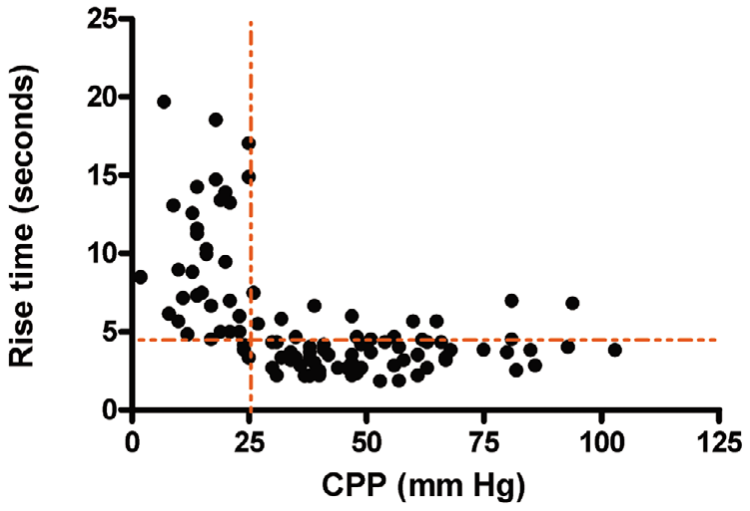
Cerebral perfusion pressure (CPP) and near infrared spectroscopy derived rise time. *n* = 76 measurements in 20 animals.

**Figure 8:**
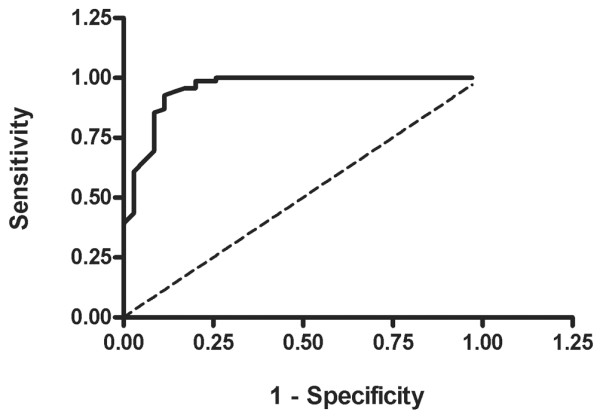
Receiver-operator curve using near infrared spectroscopy derived interval time for a threshold of cerebral perfusion pressure <25 mmHg. Area = 0.95 (95% confidence interval 0.92 to 0.99), *p* < 0.0001.

Analyzing interval and rise time, there was a threshold below a CPP of 25 mmHg (Figures 6 and 7). Receiver-operator curves were calculated for both parameters (Figure 8). An interval time >8 seconds yielded an 84% sensitivity and a 91% specificity to indicate a CPP below 25 mmHg. For a rise time of >4.7 seconds, the respective values were 83% and 89%, and the area under the ROC curve was 0.93 (95% confidence interval 0.89 to 0.98, *p*< 0.0001).

Arterial partial oxygen pressure (PaO_2_) values closely reflected changes in FiO_2_, and arterial CO_2 _tension (PaCO_2_) changes were related to CO. Overall, resuscitation was successful in 15 out of 20 animals.

## Discussion

The main findings of the present prospective experimental study are as follows. First, at CPP below 20 mmHg during hemodynamic decompensation, both BFI and TCD suggested a significantly reduced CBF. Second, after resuscitation, both parameters reached approximately baseline values. Third, BFI and TCD were significantly correlated with each other as well as with CPP and CO. Fourth, both interval and rise time markedly increased below a CPP of 25 mmHg and may be sensitive and specific parameters in this respect.

During past decades, reliable monitoring of cerebral perfusion has been challenging. General parameters, such as cardiac output and blood pressure, are normally not sufficient to provide information in this respect, since brain circulation is controlled by autoregulation, and cerebral pathology can impair cerebral perfusion despite an intact systemic circulation [11].

TCD is a non-invasive method of determining beat-by-beat relative changes in cerebral blood flow velocity, which has been widely adopted for indirect measurement of CBF [12]. In a considerable proportion of subjects, however, it is not possible to obtain a signal derived from the middle cerebral artery (MCA)MCA through the temporal bone window [3]. TCD examinations require specific training and measurements may be influenced by individual skills.

NIRS has evolved as a non-invasive method to monitor cerebral oxygenation on the intact skull by measuring the different light absorption patterns of oxygenated and deoxygenated hemoglobin and calculating a regional oxygen saturation [5]. NIRS technology has advanced tremendously in recent years. Specifically, the introduction of spatially resolved spectroscopy (commercially available in the NIRO 300 used in this study) fuelled enthusiasm that the influence of extracerebral contamination could be reduced significantly [13,14]. Even with this sophisticated algorithm, however, some problems remain unresolved. For example, the exact proportion of near infrared light travelling through brain tissue is unknown, and adjustment of signals for inter-individual variation of superficial tissue thickness is, therefore, not possible [15].

More recently, detection of ICG dilution curves on the intact skull by NIRS has gained increasing attention. It has been suggested that the use of ICG may overcome the limitation of extracerebral signal contamination, since the first part of the dilution curve used for determination of ICG kinetics represents early dye arrival in the brain, which is delayed in the upper layers [16]. Furthermore, ICG detection by NIRS is not influenced by hemoglobin present outside the vascular bed (for instance after intracerebral hemorrhage), since the specific extinction coefficient of ICG differs largely from that of hemoglobin at all four wavelengths applied. The BFI has been shown to reflect CBF in an animal study; there was a close correlation to cortical blood flow, but not to skin blood flow [6]. In this study, however, the lowest aortic pressure reported (48.8 ± 2.1 mmHg) did not reliably fall short of the autoregulatory threshold, which limits application of the results to patients with an impaired autoregulation or during severe hypotension. By contrast, CPP was significantly below this threshold in the present study. The significant correlation observed between both BFI and TCD with systemic hemodynamics seems counterintuitive at first, since cerebral autoregulation prevents direct coupling of cerebral and systemic circulation under normal conditions [17]. Following hemorrhage, however, CPP was markedly below the autoregulatory threshold in the vast majority of animals, even ten minutes after return of spontaneous circulation. This suggests a pressure dependent CBF in these subjects that in turn led to the observed correlation between systemic and cerebral perfusion. On the other hand, the tight correlation between measures of cerebral perfusion and CPP indicates that, at least during impaired autoregulation, CPP is indeed a valuable tool for estimation of brain perfusion. Comparably, CO was significantly correlated with both TCD and BFI. As discussed above, during the majority of measurements an impaired autoregulation is highly likely. Since CO and CPP were tightly correlated, the correlation of CO and cerebral perfusion may be explained simply by changes in CPP. Given an unchanged vascular resistance, an increasing CO will in turn increase CPP. This holds true, however, only during periods of impaired autoregulation if vascular resistance and intracranial pressure both remain unchanged. Under these circumstances, measuring CO may provide valuable information regarding cerebral perfusion.

Interestingly, BFI values showed a relatively small inter-individual variance during intact autoregulation. CV of BFI measurements between subjects was 31%, which is comparable to TCD (32%). Established methods of CBF determination may have an intra-individual CV as large as 31% [18]. Theoretically, comparison of BFI values between subjects is hampered by the fact that flow is not determined in absolute values, but with a proportionality factor. This factor may vary considerably between subjects dependent on layer thickness between NIRS optodes and the cerebral tissue interrogated. Consequently, Wagner and co-workers [7] reported a large inter-individual variability in a heterogeneous pediatric population in the ICU. The limited variance obtained in our study, however, suggests that this proportionality factor may be very similar in definite subpopulations and warrants further investigations. Furthermore, both NIRS derived time intervals showed a distinct threshold for a CPP below 25 mmHg with high sensitivity and specificity. In a recent TCD investigation during induced arterial hypotension for endovascular stent-graft placement, in 81% of patients, MAP decreased below 40 mmHg, which is comparable to the CPP threshold in the present study [19]. Although a CPP of 25 mmHg normally is beyond a critical threshold in daily clinical practice, such a pattern may serve as a wake-up call for immediate action.

BFI was significantly correlated with TCD readings. The relatively weak correlation (r = 0.62) may be explained, at least in part, by the fact that BFI represents blood flow in a small tissue sample whereas TCD measures global cerebral perfusion. NIRS is capable of interrogating tissue samples at a depth of approximately one-quarter to one-half the interoptode distance (5 cm in the present study), which limits the depth of near infrared (NIR) light penetration to roughly 1.25 to 2.5 cm, although a banana-shaped region of sensitivity extends both above and below this depth [20,21]. Kohri and colleagues [22], combining spatially and time resolved spectroscopy, estimated the contribution ratio of cerebral tissue to whole optical signals at a source detector distance of 3 cm and 4 cm as 55% and 69%, respectively. Furthermore, vessel diameter of the MCA may have changed during low-flow states. The basic assumption with TCD methodology is that relative changes in FVmean represent corresponding changes in CBF. While it has been shown in humans that MCA diameter remains unchanged during various physiological stimuli and following vasoactive drug administration [23,24], species-specific properties of vascular beds may exist and, therefore, we cannot completely rule out a change in MCA dimension in our animals [25]. Given the finite spatial resolution of the ultrasound beam, such a constricted vessel may have been missed [26], while detection of ICG passage through the cerebral vasculature is only dependent on a sufficient amount of infrared light injected into cerebral tissue. The correlation found in the present investigation applying ICG, however, is very similar to the results obtained comparing TCD with NIRS derived oxygenation parameters in patients and healthy volunteers during vasomotor reactivity tests [3,14].

Some limitations of our study should be noted. ICG derived NIRS measurements need specific training, which mainly relates to setup of the monitor in the ICG mode and appropriate timing and speed of ICG injection. Therefore, ICG was always injected by the same investigator. BFI was obtained only once at the different experimental stages in each animal. This, however, does not introduce a considerable random error, since a high intra-individual reproducibility of BFI measurements was demonstrated recently during repeated measurements in pediatric patients [7]. ICG is eliminated by biliary excretion and during hypovolaemic shock, liver blood flow is significantly decreased. However, BFI determination is robust against residual plasma ICG. This is rooted in the fact that continuous detection of ICG absorption by NIRS clearly indicates that plasma ICG is not adequately removed. More important, the BFI equation subtracts baseline value from peak value. Therefore, the calculation algorithm accounts for any residual ICG. In our study, however, baseline was approximately zero for all measurements.

Animals were treated with either arginine vasopressin or norepinephrine for resuscitation, and potentially both drugs exert direct and different effects on the large cerebral vessels insonated by TCD, thereby influencing correlation with BFI. It has been shown, however, that both drugs do not influence diameter of large cerebral vessels significantly [24,27]. Furthermore, this limitation applies only after drug administration. Since all TCD examinations were performed by the same experienced physician, differences regarding inter-individual skills did not influence TCD readings.

The path length of NIRS in the porcine head is unknown and thus we used the published data derived from human experiments [28]. The porcine skull is most similar to humans in the frontal and periorbital region, both with respect to skull structure and thickness of the overlying skin. Although there may be a difference between species, this does not interfere with the results of our study since it was aimed at investigating correlation between TCD and BFI rather than presenting absolute values.

## Conclusion

The results of the present study show that both BFI and TCD reflected the large changes in cerebral perfusion provoked by hemorrhage and were significantly correlated. ICG derived BFI is a promising method for non-invasive determination of cerebral hemodynamics over a wide range of flow conditions. BFI may be advantageous in patients where an ultrasound signal of sufficient quality is difficult to obtain. The definition of threshold values for BFI and ICG derived time intervals in specific patient populations and under differing flow conditions is warranted.

## Key messages

• 	BFI and TCD both reflect large changes in cerebral hemodynamics.

• 	BFI and TCD are significantly correlated.

• 	BFI is a promising method for non-invasive determination of cerebral hemodynamics over a wide range of flow conditions.

• 	Further studies for definition of threshold values for BFI in specific patient populations and under differing flow conditions are warranted.

## Abbreviations

BFI = blood flow index; CBF = cerebral blood flow; CO = cardiac output; CPP = cerebral perfusion pressure; CV = coefficient of variation; FiO_2 _= fraction of inspired oxygen; FVmean = mean blood flow velocity; ICG = indocyanine green; ICP = intracranial pressure; ICU = intensive care unit; MAP = mean arterial pressure; NIRS = near infrared spectroscopy; PAC = pulmonary artery catheter; TCD = transcranial Doppler ultrasound.

## Competing interests

The authors declare that they have no competing interests.

## Authors' contributions

BB performed data acquisition and analysis of the near infrared spectroscopy derived data and drafted the manuscript. PM performed the transcranial Doppler studies and analysed TCD data. EC carried out anesthesia and instrumentation of the animals and was responsible for hemodynamic data. PHT made a significant contribution to drafting the manuscript (Discussion section). MS performed the laparotomy. JS participated in the study design and helped to draft the manuscript. VD conceived of the study and helped to draft the manuscript (Methods section). All authors read and approved the final manuscript.

## Figures and Tables

**Table 1:** Hemodynamic data, blood gases and NIRS values at the different experimental stages

Hemodynamic variables and blood gases	Baseline (*n* = 20)	Baseline therapy (*n* = 20)	Therapy + 10 min (*n* = 19)	Therapy + 40 min (*n* = 15)	Therapy + 90 min (*n* = 15)
		
CPP (mmHg)	70 ± 16	16 ± 5^a^	34 ± 16^a,b^	43 ± 16^a,c^	46 ± 20^a,c^
HR (beats·min^-1^)	99 ± 22	193 ± 27^a^	194 ± 30^a^	202 ± 32^a^	200 ± 35^a^
CO (l·min^-1^)	5.8 ± 1.3	2.0 ± 0.5^a,d^	2.9 ± 0.7^a,d^	3.6 ± 1.3^a,b,d^	6.2 ± 2.0
ICP (mmHg)	14 ± 4	9 ± 3^a^	9 ± 3^a,e^	11 ± 2	13 ± 2^b^
PaO_2 _(mmHg)	152 ± 36	141 ± 19	434 ± 45^a,c,d^	230 ± 27^a,c,f^	233 ± 16^a,c^
PaCO_2 _(mmHg)	41 ± 5	35 ± 3^a^	48 ± 4^a,c^	51 ± 4^a,c^	48 ± 4^a,c^
		
NIRS variables after ICG injection	*n* = 18	*n* = 18	*n* = 17	*n* = 13	*n* = 10
		
Time interval (0–100%; s)	7.5 ± 1.1	16.7 ± 5.4^a^	7.6 ± 2.2^c^	5.7 ± 1.4^c^	5.1 ± 1.4^c^
Rise time (10–90%; s)	4.4 ± 1.1	11.2 ± 3.9^a^	4.5 ± 2.1^c^	3.1 ± 1^c^	3.0 ± 1.0^c^
Slope (μmol·l^-1^·s^-1^)	0.012 ± 0.003	0.005 ± 0.002^a^	0.009 ± 0.004^e^	0.012 ± 0.005^c^	0.014 ± 0.007^c^

Data are given as mean ± standard deviation. ^a^*p* < 0.001 versus baseline; ^b^*p* < 0.01 versus baseline therapy; ^c^*p* < 0.001 versus baseline therapy; ^d^*p* < 0.001 versus therapy + 90 minutes; ^e^*p* < 0.05 versus therapy + 90 minutes; ^f^*p* < 0.001 versus therapy + 10 minutes. CO, cardiac output; CPP, cerebral perfusion pressure; HR, heart rate; ICP, intracranial pressure; ICG, indocyanine green; NIRS, near infrared spectroscopy; PaCO_2_, arterial CO_2 _tension; PaO_2_, arterial partial oxygen pressure.
